# Peripheral blood lymphocyte subsets in children with nephrotic syndrome: a retrospective analysis

**DOI:** 10.1186/s12882-022-03015-y

**Published:** 2023-01-10

**Authors:** Yan Deng, Ying-ying Ou, Cui-Ju Mo, Li Huang, Xue Qin, Shan Li

**Affiliations:** Key Laboratory of Clinical Laboratory Medicine of Guangxi Department of Education, Department of Clinical Laboratory, First Affiliated Hospital of Guangxi Medical University, Nanning, 530021 Guangxi China

**Keywords:** Nephrotic syndrome, Children, Lymphocyte subset, Immunosuppressive treatment

## Abstract

**Background:**

Nephrotic syndrome (NS) in children is widely believed to be associated with severe changes in the immune system. Based on lymphocyte subset analysis, we examined the pathogenesis of immune deficiencies in children with NS with varying steroid sensitivity.

**Methods:**

Our study utilized flow cytometry to retrospectively analyze the ratios of lymphocyte subsets in 204 children with nephrotic syndrome and 19 healthy children.

**Results:**

Compared with healthy children, the ratio of CD4 + /CD8 + in onset and remission was decreased in SRNS group (*p* < 0.05), and CD19 + B lymphocytes were increased in onset (*p* < 0.05). Compared with onset, the proportion of CD19 + B lymphocytes decreased in SRNS, while the proportion of CD19 + B lymphocytes increased in SDNS, *p* < (0.01). The ratio of CD8 + T/CD19 + B in onset in SDNS group was significantly higher than that in SSNS and SRNS groups (*p* < 0.01) and healthy control group (*p* < 0.05). Compared with onset, the ratio of CD8 + T/CD19 + B in SDNS group decreased significantly (*p* < 0.01), while the ratio of CD8 + T/CD19 + B in SRNS group increased significantly (*p* < 0.01). The proportion of CD56 + CD16 + NK cells was significantly reduced in children with INS (*p* < 0.01).

**Conclusion:**

CD8 + T lymphocytes may be involved in the mechanism of lymphocyte subsets disorder during onset of SDNS, while CD19 + B lymphocytes may be involved in the mechanism of lymphocyte subsets disorder during relapse of SDNS. The CD8 + T/CD19 + B ratio may predict the degree of frequent recurrence. There is a certain degree of lymphoid subsets disorder in children with NS.

## Introduction

The Idiopathic Syndrome (INS) is a common childhood kidney disease caused by a ruptured capillary wall that allows large molecules to leak from the blood into the urine [1, 2]. The reported incidence of NS in children is 4.7 per 100,000 children worldwide (range: 1.15–16.9) [3]. The prevalence of INS is highest among children between the ages of 2 and 7, with the majority of cases occurring in boys (3.8 boys per girl). Currently, the pathologic process of INS is not clear. However, a large number of previous studies have found that the abnormality of immune cells and disorders in immune function are related to INS [3, 4].

 A course of oral corticosteroids is the initial treatment for INS, and the prognosis is determined by the response to corticosteroid therapy [5]. Clinical classification supports a response to corticoid medical care. 80% to 90% of children one year old or older with INS respond to steroid treatment within four weeks [steroid-sensitive syndrome (SSNS)], whereas the remaining 10%–20% do not respond, exhibiting a condition known as steroid resistant nephrotic syndrome (SRNS). Even in children with SSNS who are sensitive to steroid medical aid, the subsequent course of the disease varies greatly, with most children having a minimum of one relapse. Most children have frequent repeated nephrosis (FRNS) (two or more recurrences in the initial half-dozen months or four recurrences a year) or steroid-dependent nephrosis (SDNS) (recurrences within two weeks of ceasing steroid therapy or during treatment with steroids) [6].

Previous studies have indicated that the most pathological process of INS is caused by T lymphocytes dysfunction or abnormal secretion of a capillary porousness factor [7]. Studies have found that peripheral blood CD3 + , CD4 + , CD4 + /CD8 + and IgG/IgM are decreased in children with NS [4]. Other recent studies indicate that the pathological process of INS might also be associated with the abnormal performance of B lymphocytes [8]. Chen et al. [9] found that B cell subsets are involved in the pathogenesis of SSNS.

To assess the sensitivity of children to steroids and effectively intervene early, we hoped to find specific biological indicators in children with INS, which would aid in the prognosis of children and cut down the recurrence rate [4]. Therefore, we studied the expression of peripheral leukocyte subsets, immunoserum globulin, and complements in children with SSNS, SRNS, and SDNS/FRNS, hoping to provide new ideas for clinical treatment.

## Materials and methods

### Patients and healthy individuals

In this study, 204 children (179 boys and 25 girls with an age range of 12–168 months) were included. The control group included 19 healthy children (15 boys and 4 girls with an age range of 48–132 months). A classification of children with NS was made after the initial diagnosis based on their sensitivity to steroids:27 children with SSRS (complete response after 4 weeks of corticosteroid treatment), 52 children with SRNS (no complete response after 8 weeks of corticosteroid treatment), and 45 children with SDNS (two consecutive relapses during steroid therapy or within 14 days of the end of treatment) [6]. Based on age- and gender-matched INS onset groups, remission children (RS microalbuminuria or negative proteinuria) were divided into three remission groups: SSRS remission (19 cases), SRNS remission (13 cases), and SDNS remission (48 cases). The laboratory results were subsequently recorded. Exclusion criteria were applied in the following cases: (1) the children were younger than 1 year or older than 14 years; (2) there was a positive family history of nephropathy; (3) the children had a malignant tumor; (4) the children had congenital immunodeficiency; (5) the children had other autoimmune diseases, such as lupus and purpura; and (6) the children had incomplete clinical data.

### Experimental data collection

The relevant indicators of the patients were observed. All indicators were detected using the corresponding instruments and matching kits. Serum albumin (ALB), serum creatinine (SCr), and endogenous creatinine clearance (Ccr), as well as IgA, IgG, IgM, and complement C3 and C4 levels, were measured using an automated biochemical analyzer (Abbott C16000). A Beckman whole blood cell analyzer (LH750) was used to detect white blood cells (WBC), platelets (PLT), neutrophils (NEU), lymphocytes (LYM), and monocytes (MON). The peripheral blood was tested with flow cytometry (BD Canto II) for the detection of CD3 + T, CD3 + CD4 + T, CD3 + CD8 + T, CD19 + B, and NK subsets.

### Statistical analysis

 The Kolmogorov-Smirnov test was used to determine the normality of continuous data. The means, standard errors, and medians were presented as means + standard errors. Continuous variables were compared using a parametric ANOVA or nonparametric Kruskal–Wallis test, and pair-to-pair differences were compared using an unpaired T test or Mann–Whitney U test, respectively. In addition to constructing a receiver operating characteristic (ROC) curve and determining the area under the curve (AUC) to gauge the prognosticative strength, it was also determined the optimal critical point to maximize its sensitivity and specificity. All *P* values were bilateral, and statistical significance was set as *p* < 0.05.

## Results

### Population, statistical, and clinical characteristics of the children and healthy controls

Table 1 lists the demographic characteristics of the listed subjects, i.e., 204 children with nephrotic syndrome. A total of 124 patients were classified according to their response to prednisone at onset prior to any immunosuppressive therapy (SSNS = 27, SRNS = 52, SDNS/FRNS = 45), and 80 patients were classified into the stable remission stage (SSNS remission = 19, SRNS remission = 13, SDNS/FRNS remission = 48; the stable remission group matched the onset group for age and sex). Between onset and remission groups, as well as between onset and remission groups and healthy controls, there was no difference in age or sex. Compared with the remission group and healthy children, the albumin level in every INS cluster was lower, the globulin/albumin quantitative relation was lower, and the ChE and sterol levels were higher. ChE was positively correlated with serum cholesterol, significantly negatively correlated with IgG, and positively correlated with IgM, C3, and endogenous creatinine clearance. At onset and during remission, serum creatinine (G/L) levels were significantly reduced in each INS group. In the SRNS group, serum globulin levels decreased during onset and remission, while Ccr levels were higher in the SSNS and SDNS groups.

**Table 1:**
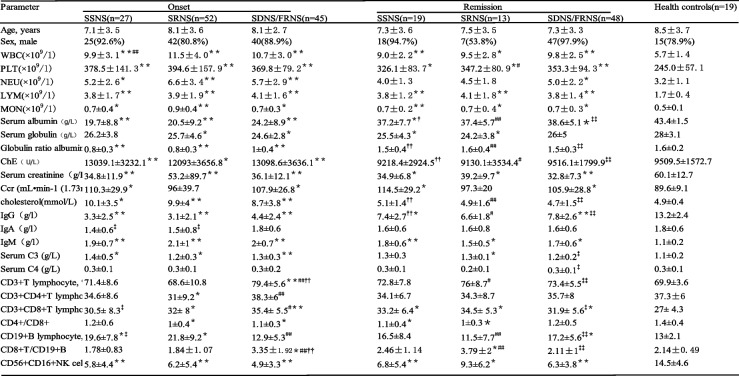
Demographic and clinical characteristics of patients with INS

*SSNS* Steroid sensitive, *SRNS* Steroid resistant, *SDNS/FRNS* Steroid dependent, *IG* Immunoglobulin, *%L* Percentage of lymphocytes

^#^*p* < 0.05; ##*p* < 0.01, compared to SRNS patients at onset. †*p* < 0.05; ††*p* < 0.01, compared to SSNS patients in onset

^‡^*p* < 0.05; ‡‡*p* < 0.01, compared to SDNSpatients in onset. *p < 0.05; **p < 0.01, compared to controls

### Peripheral blood cells, antibody protein, and complement analysis of INS patients

In all NS groups, WBC, PLT, NEU, LYM, and MON levels were higher than in healthy children. For WBC levels, those in the SRNS group were significantly higher than those in the SSNS group. PLT levels and LYM and MON ratios were significantly increased in each NS group at onset and remission. The proportion of NEU in the SSNS and SRNS groups was lower in remission than at onset. The IgG levels of each INS group were significantly lower than those of the control group and the remission group, and the IgM levels were significantly higher than those in healthy children. Children with SSNS and SRNS had lower IgA levels than healthy children. The complement C4 levels in all groups did not differ significantly, while complement C3 levels in the INS incidence groups were higher than those in the healthy control group, and SDNS/FRNS decreased in remission.

### Lymphocyte subsets of children with INS

**Fig. 1:**
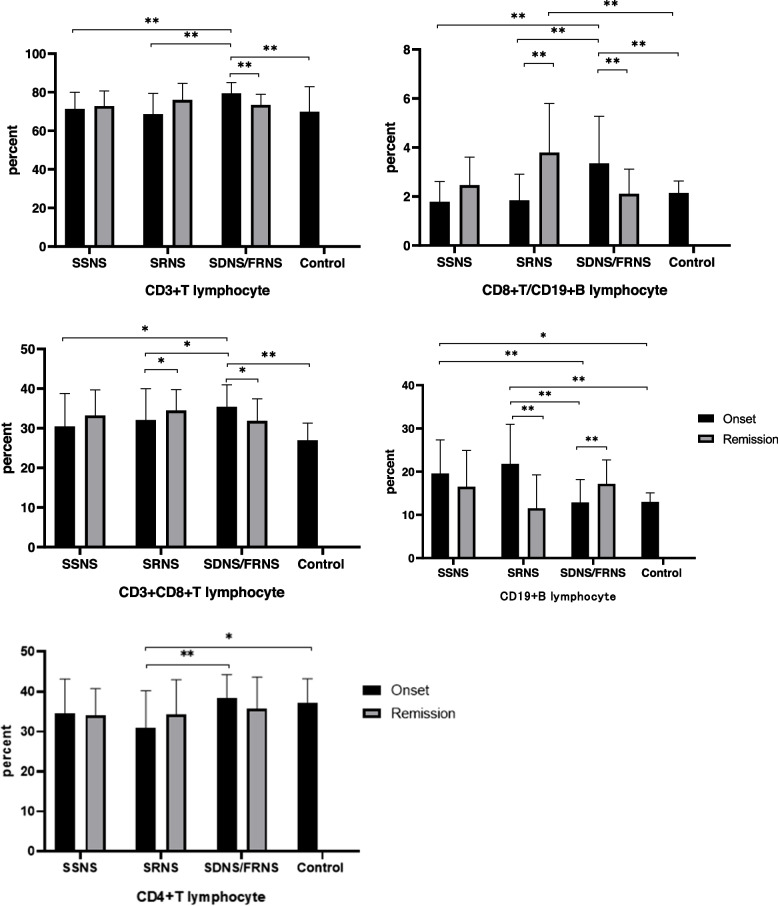
Flow cytometry analysis of peripheral blood white blood corpuscle subsets in children with INS.* *P* < 0.05, *P* < 0.01. ** They are expressed as a percentage of the total number of lymphocytes

The SDNS/FRNS groups had a significant increase in CD3 + T lymphocyte proportions, especially in CD4 + T and CD8 + T lymphocytes, when compared to the SSNS and SRNS groups. The proportion of CD3 + T and CD8 + T lymphocytes decreased during remission (Fig. 1). CD4 + /CD8 + was lower in the SRNS group at the onset and remission stages. SRNS increased CD19 + B lymphocytes and decreased in remission, whereas SDNS/FRNS increased in remission. The CD8 + T/CD19 + ratio increased in the SDNS/FRNS group and decreased in remission, while SRNS increased in remission. The proportion of CD56 + CD16 + NK cells was significantly decreased in each NS group.

### Predictive diagnostic analysis of lymphoid subsets

**Fig. 2:**
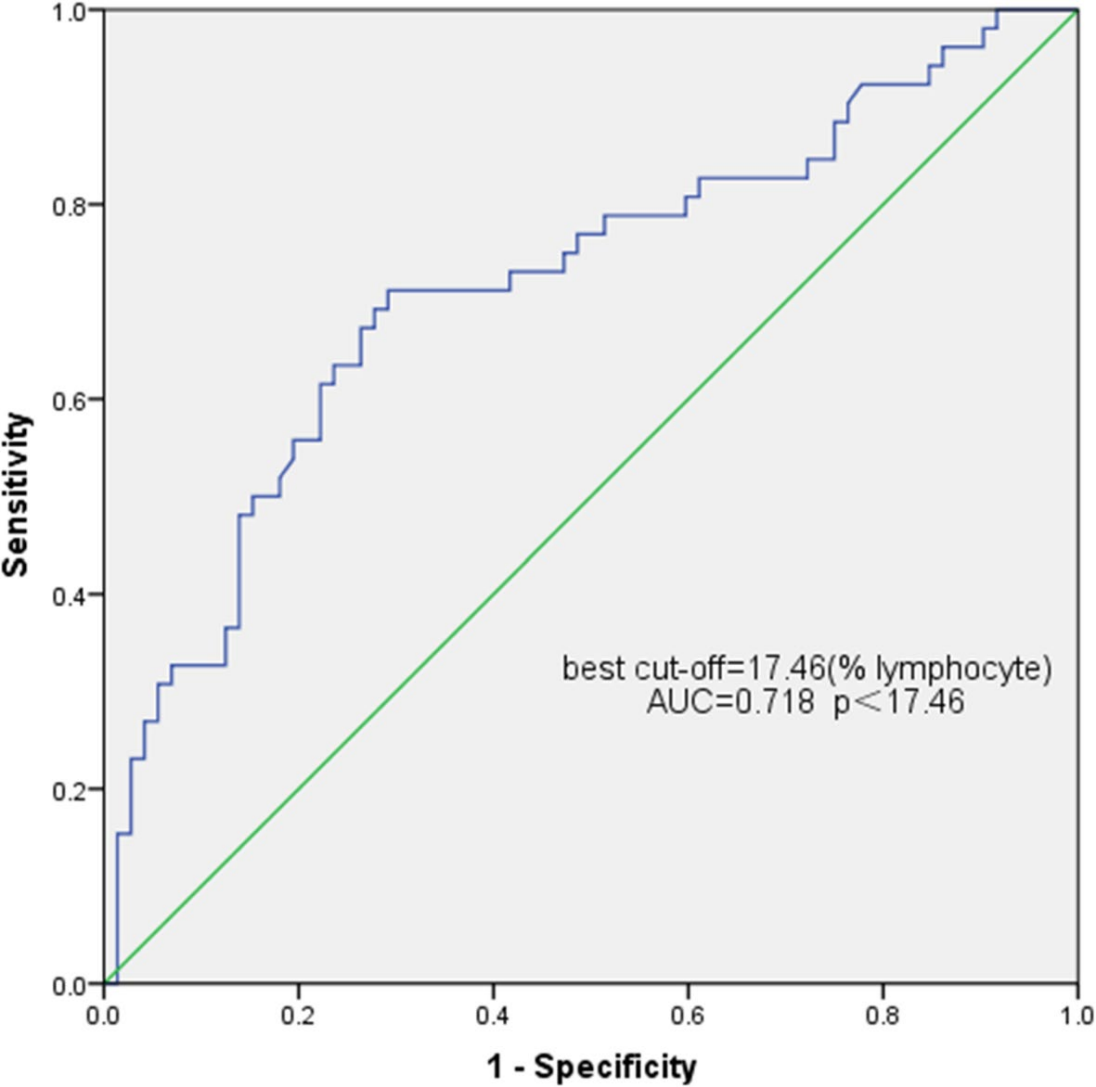
Receiver operating characteristic curve analysis of CD19 + B cells as markers of SRNS recognition

**Fig. 3:**
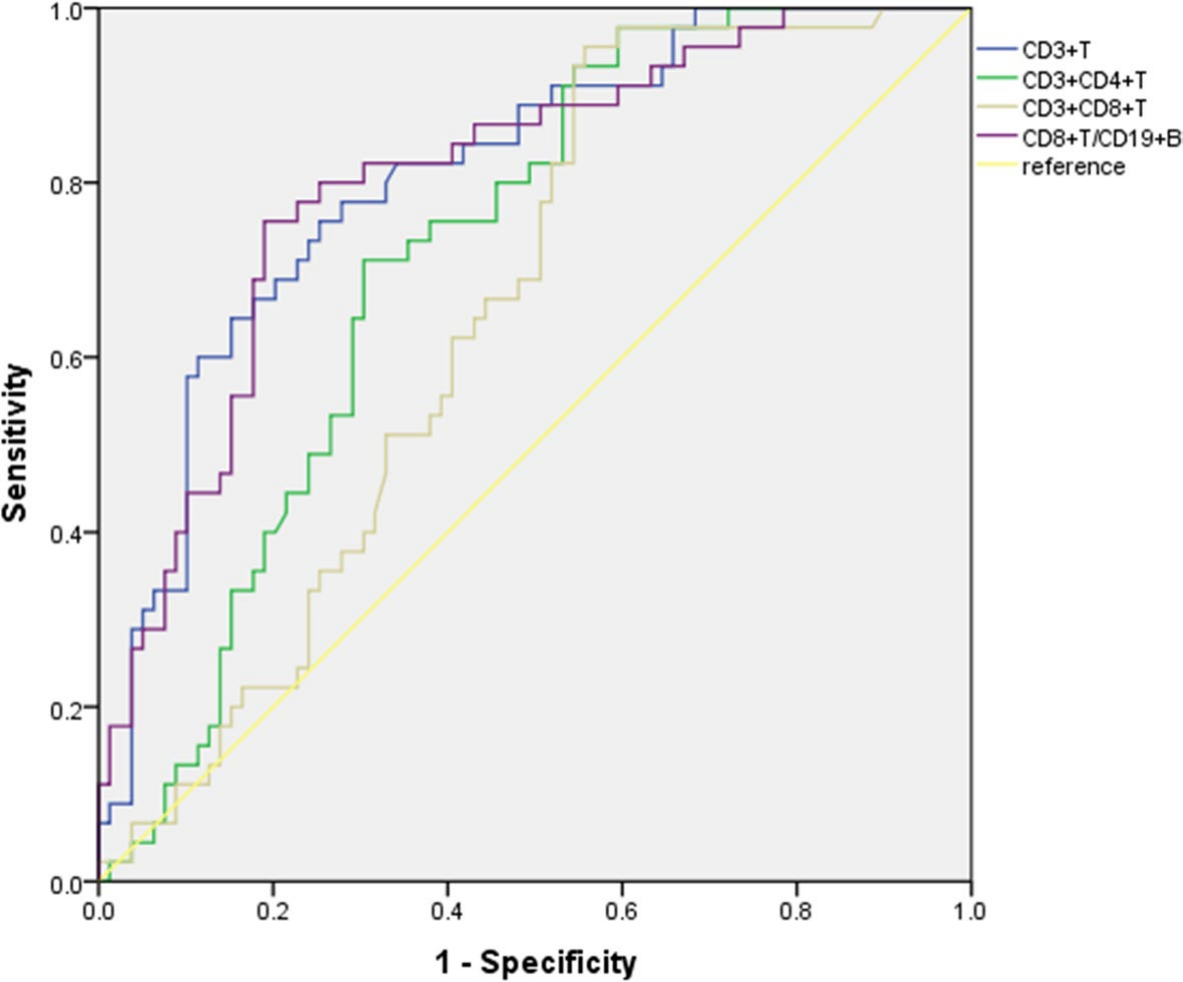
Receiver operating characteristic (ROC) analysis showing the overall accuracy of different T-lymphocyte subsets in SDNS patients

We attempted to use lymphocyte subsets to predict the outcome of corticosteroid therapy in the diagnosed SSNS, SRNS, and SDNS/FRNS patients. ROC curves were used to assess the potential utility of CD19 + B lymphocytes at the onset of SRNS. The area under the ROC curve for CD19 + B lymphocytes to predict SRNS was 0.718 (95% confidence interval, 0.624–0.812). The optimal cut-off value was 17.46%/L, corresponding to 71.2% sensitivity and 70.8% specificity (Fig. 2). The ROC curves of CD3 + T, CD3 + CD4 + T, CD3 + CD8 + T, and CD8 + T/CD19 + B were used to predict and evaluate the SRNS/FRNS children (Fig. 3, Table 2).

**Table 2:**
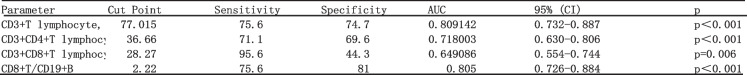
Sensitivity, specificity, and accuracy of T lymphocyte subsets and CD8 + T/CD19 + B in predicting SDNS patients

## Discussion

A large amount of data indicates that T cells and B cells are major participants in the pathogenesis of INS. The dysfunction or abnormal regulation of T lymphocytes is related to the pathogenesis of INS [7]. CD4 and CD8 cells play an important role in aiding and regulating immunity by releasing cytokines and other inflammatory mediators, which lead to renal injury. INS diseases may also develop due to an imbalance between CD4 and CD8, which affects immune function [10, 11]. Compared with healthy children, the proportion of CD4 + T cells expressing the inflammatory cytokine TNF-α is increased in children with INS, and the proportion of CD8 + T cells expressing IFN-γ is lower [12]. However, glucocorticoids and cyclosporine do not completely inhibit the expression of T-lymphocyte inflammatory cytokines, which may contribute to persistent or frequent recurrence of symptoms and ultimately to inadequate sensitivity to steroids [12]. Furthermore, the results of the study revealed an unbalanced distribution of CD4/CD8 T lymphocytes, which was conducive to recurrences and remissions of CD8 T lymphocytes [13]. CD8 T cells could be cloned and amplified during continuous NS disease activity [14]. As a result of our study, we found that the proportion of T cells in SRNS children did not significantly differ from that in the control group. However, CD8 increased and CD4 and CD4/CD8 decreased in the SRNS children, indicating that the disorder of T lymphocyte subsets occurred in the SRNS children at the onset. The disorder and abnormal regulation of CD4 and CD8 may make children less sensitive to steroid therapy. We found that the proportion of total T lymphocytes in onset increased in SDNS children compared with the other two groups of children and healthy children, especially the proportion of CD8 + T significantly increased in Onset, and decreased in remission. This suggests that CD8 + T may be involved in the pathogenesis of SDNS. After systematic steroid treatment, the proportion of T lymphocytes in all three groups fell to the normal range. These findings are consonant with previous studies suggesting that the abnormal regulation of T cells plays a part in the pathological process of NS [15]. Notably, SSNS and SRNS groups had an increase in CD8+T, whereas SDNS had a decrease in CD8+ T. After systematic treatment, all three groups had high CD8 + T proportions, but the SRNS group had the highest CD8 + T proportion. Moreover, lymphocyte counts and T cell subsets can be monitored in patients treated with methylprednisolone to estimate their susceptibility to infection [16].

Research has shown that peripheral blood B cell counts and activation levels may influence INS disease occurrence. Compared with the HC group, transitional B cells were significantly increased in the early stage of the disease; some were regulatory B (Breg) cells, which play an immunomotor role by secreting IL-10, IL-35, and transforming growth factor β (TGF-β) [14]. Changes in B cell subsets can indirectly affect T cell subsets in lymphoid follicles that interact with B cells [17]. Transitional B cells can inhibit Th17 cell polarization [18], promote the induction of Treg cells [19], and reduce the Th1 cell secretion of IFN-γ and TNF to regulate T cell activation [20, 21]. Although considered non-pathogenic cells, abnormal transitional B-cell regulation may lead to the involvement of these T-lymphocyte subpopulations in the pathogenesis of various autoimmune nephropathy [9]. Additionally, B cell-associated depletion therapy has beneficial effects that support the role of B cell abnormalities in the pathogenesis of INS, and rituximab may correct imbalances in Treg cell to Th17 cell ratios and CD4 + T cells to CD8 + T cells by increasing dendritic cell levels [22]. We found that the total B lymphocyte ratio of SSNS and SRNS was significantly higher, and the B lymphocyte ratio of SRNS patients was significantly higher than that of the other three groups.With a cutoff value of 17.46%/L (lymphocyte), CD19 + B cells enabled the observer to predict steroid responses in SRNS patients using the AUC with high sensitivity and acceptable accuracy. This suggests that B cells may be biomarkers for distinguishing SRNS. In remission, the proportion of CD19 + B cells in SSNS and SRNS was reduced, and the proportion of B lymphocytes was reduced to the normal range, which is consistent with the findings of Chen Ling et al. [9] and Colucci [8] Interestingly, unlike the onset of SSNS and SRNS, the proportion of B lymphocytes in SDNS at the onset of disease was insignificant in relation to the control group but significantly increased in remission. After SDNS onset, the total proportion of T cells increased significantly, especially CD8 + T cells. SDNS in the remission period had a significant increase in CD19+ B lymphocytes compared with the onset period. We hypothesized that CD8 + T lymphocytes and CD19 + B lymphocytes may be involved in the mechanism of frequent recurrence of SDNS. Consistent with this observation, Kemper et al. [23] demonstrated an amplification of CD8 + T cells in NS patients within one year after disease recurrence. In addition, it has been suggested that expansion of class-switched B cells is associated with NS recurrence [24]. Our views support those of the above studies. According to Antonella [25], CD8 + T cells negatively correlated with CD19 + B cells throughout sepsis, and they were linked by a new absolute ratio (CD8/CD19 ratio), which defined the derivative immunity index IPP, suggesting that patients who had a ratio > 2.2 were immune-protective. We attempted to analyze differences between the INS incidence groups using CD8/CD19, and found that using the 2.22 cutoff, the CD8 + T/CD19 + ratio enabled the observer to distinguish SDNS children with the AUC during disease activity. Total CD3 + T, CD3 + CD4 + T, and CD3 + CD8 + T were also used to predict the likelihood of frequent recurrence, but these findings need to benefit from validation in a larger cohort. However, much analysis is required to determine whether their presence is directly or indirectly associated with the pathologic process of INS.

We found that NK cells in patients with nephrotic syndrome were changed, and the CD56 + CD16 + NK cells in INS were significantly reduced, which was consistent with Ye et al. [14]. Therefore, we believe that CD56 + CD16 + NK cells have a more lasting effect on the pathogenesis of INS. According to the amount of CD56 on the surface of NK cells, they were divided into two types: bright CD56 NK cells and dim CD56 NK cells [26]. These two NK cells have completely different immune functions. Approximately 90% of peripheral NK cells are dim CD56 NK cells, which are extremely cytotoxic. However, bright CD56 NK cells are mainly found in body fluid nodes and exert immunomodulatory functions by releasing certain cytokines and responding to other cytokines. They account for about 5–15% of peripheral NK cells [27]. In addition to the reduction of dim CD56 NK cells, Ye et al. found that bright CD56 NK cells were also increased. Nephrotic syndrome may also be associated with an imbalance between bright and dim CD56 NK cells [14].

This study has several limitations. First, this is a retrospective analysis with a small sample, which may be affected by result bias and confounding factors. The conclusion needs to be confirmed by a diversified study with a large sample. Second, we could not further explore the relationship between T, B, and NK cell subsets, clinical indicators, and steroid sensitivity, resistance, and dependence. Third, the children in onset and remission were different in each NS subgroup, which may be biased by other confounding factors. However, we did find some meaningful changes in INS, and we believe that our study lays the foundation for further research.

## Conclusion

By analyzing lymphocyte subsets, we found that a more severe lymphocyte subset disorder and abnormal regulation existed in children with SRNS, which may affect the treatment and prognosis of corticosteroids. Compared with SRNS, SDNS showed a greater increase in CD8+ T lymphocytes at onset, whereas B lymphocytes increased mainly in remission. Therefore, we hypothesized that CD8 + T and B lymphocytes might be involved in the mechanism of the frequent recurrence of SDNS. In summary, patients with nephrotic syndrome have impaired immune performance mediated by abnormal cells, together with T cells, B cells, monocytes, and NK cells. Immune factors are also involved in the pathological process of nephrotic syndrome. We used CD3 + T, CD3 + CD4 + T, CD3 + CD8 + T, CD19 + B, and CD8 + T/CD19 + B lymphocytes to predict the degree of frequent recurrence and drug resistance to provide a better basis for clinical practice and monitor the immune status of children.

## Data Availability

We state that materials described in the manuscript, including all relevant raw data, will be freely available from the corresponding author to any scientist wishing to use them for non-commercial purposes, without breaching participant confidentiality.
